# Supplementary material for: Angiotensin converting enzyme 2 is a novel target of the γ-secretase complex
Source: Sci Rep. 2021 May 7;11:9803. doi: 10.1038/s41598-021-89379-x (PMC8105332; doi:10.1038/s41598-021-89379-x)
Supplement: Supplementary file 1 — Supplementary Figures. [file 41598_2021_89379_MOESM1_ESM.pdf]

Supplementary information

Angiotensin converting enzyme 2 is a novel target of the γ-secretase complex

Alberto Bartolomé[1], Jiani Liang[1], Pengfei Wang[2], David D. Ho[2] and Utpal B. Pajvani[1*]

[1] Department of Medicine, Columbia University Irving Medical Center, New York, NY, USA.

[2] Aaron Diamond AIDS Research Center, Columbia University Irving Medical Center, New York, NY, USA.

* Corresponding author: Utpal B. Pajvani

Email: up2104@cumc.columbia.edu

Fig1B

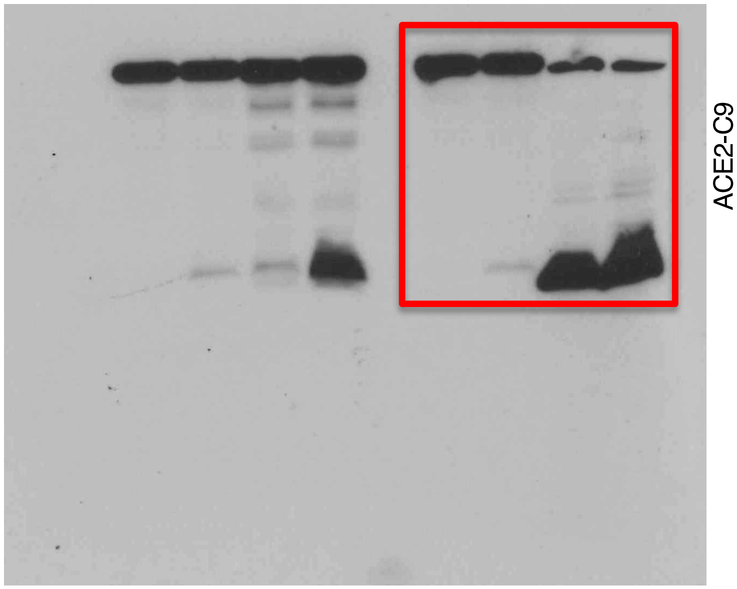

ACE2-C9

Hi exp.

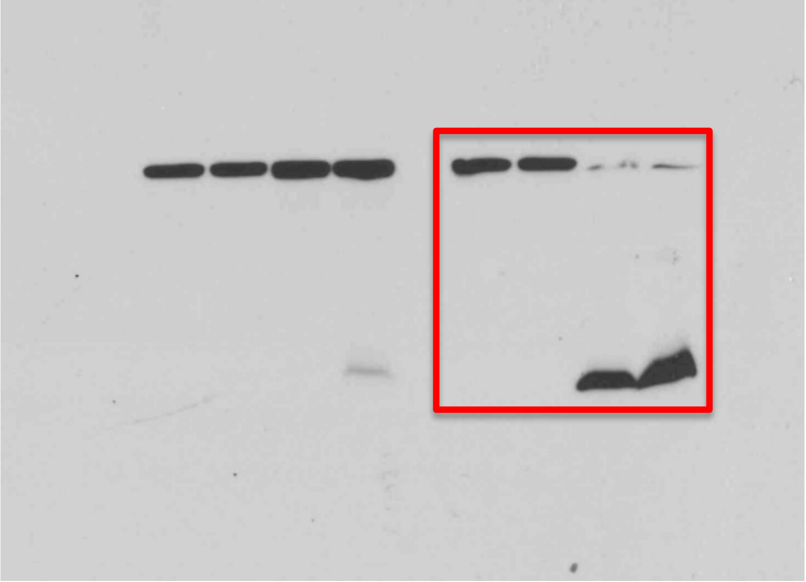

ACE2-C9

β-actin

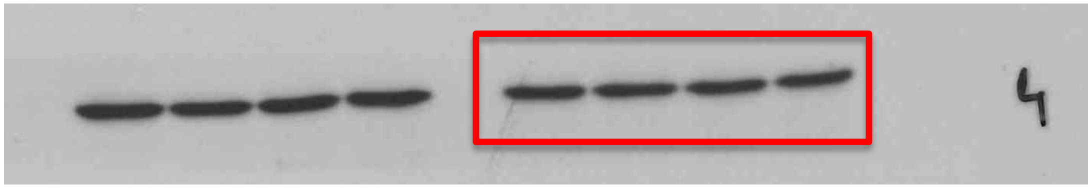

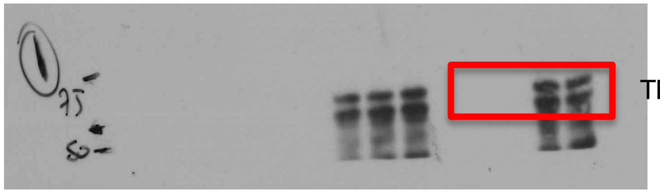

TMPRSS2

Fig1C

ACE2-C9

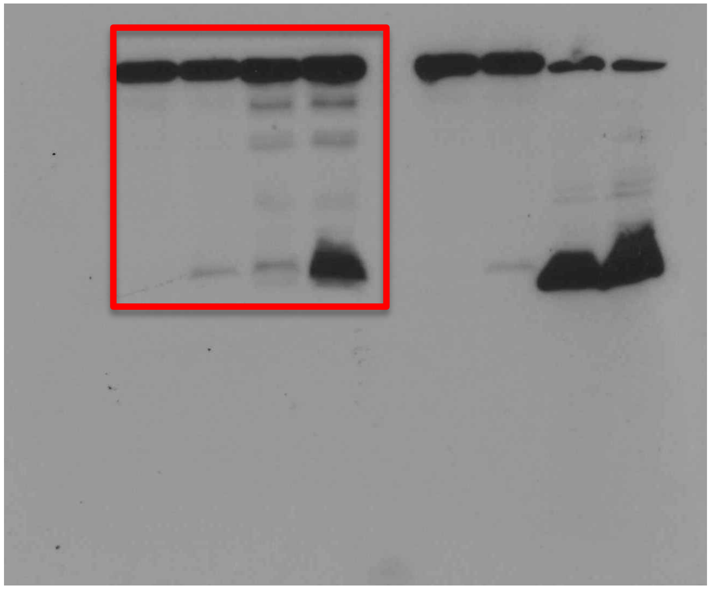

β-actin

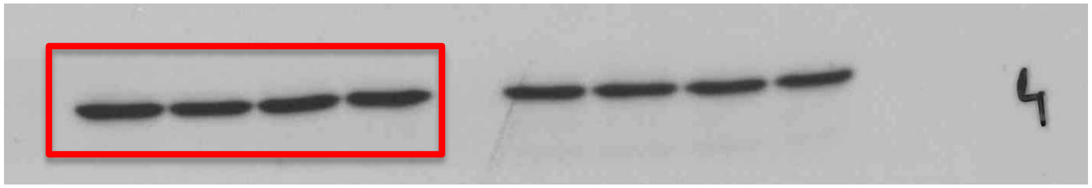

Fig1D

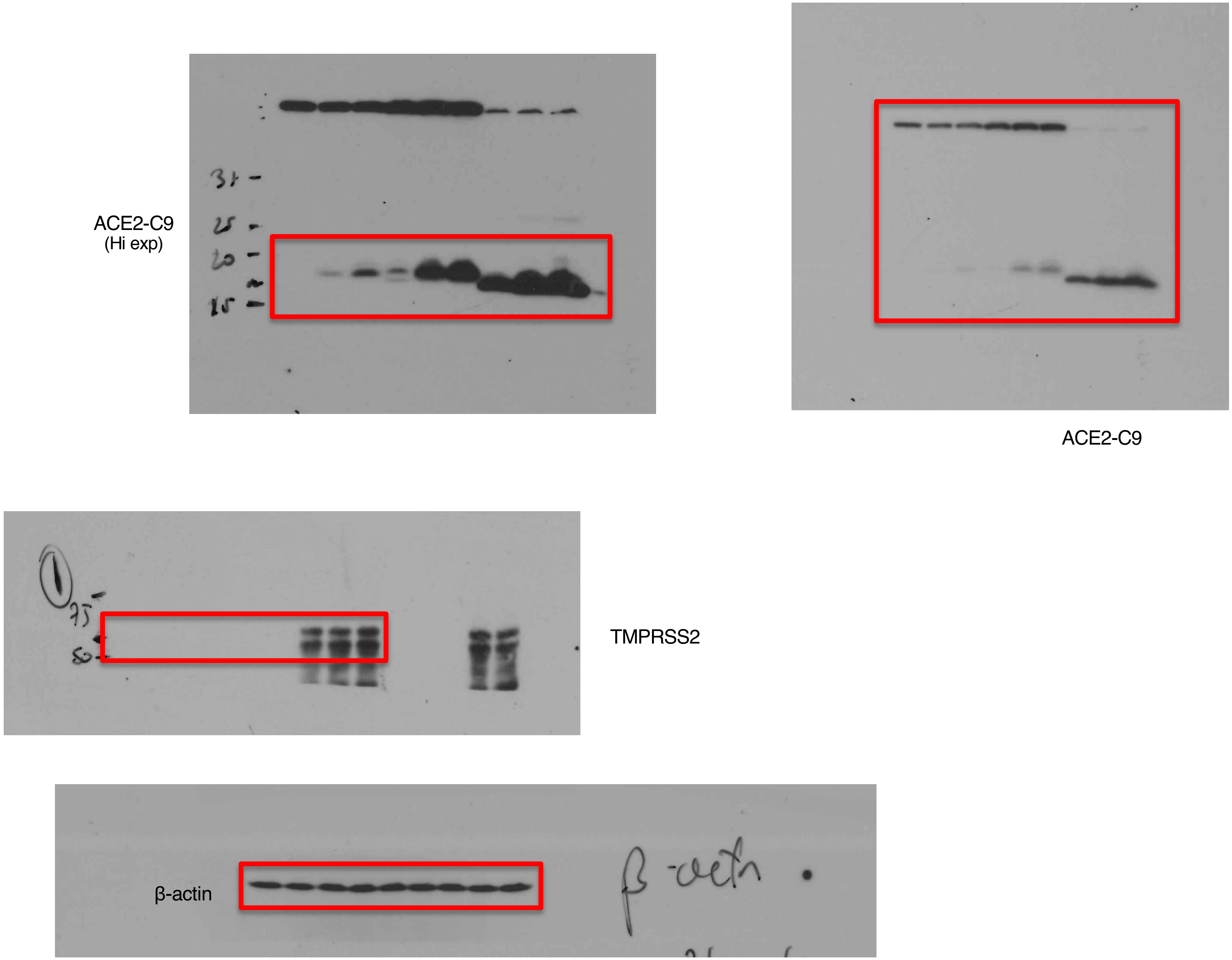

Fig1E

ACE2-GFP

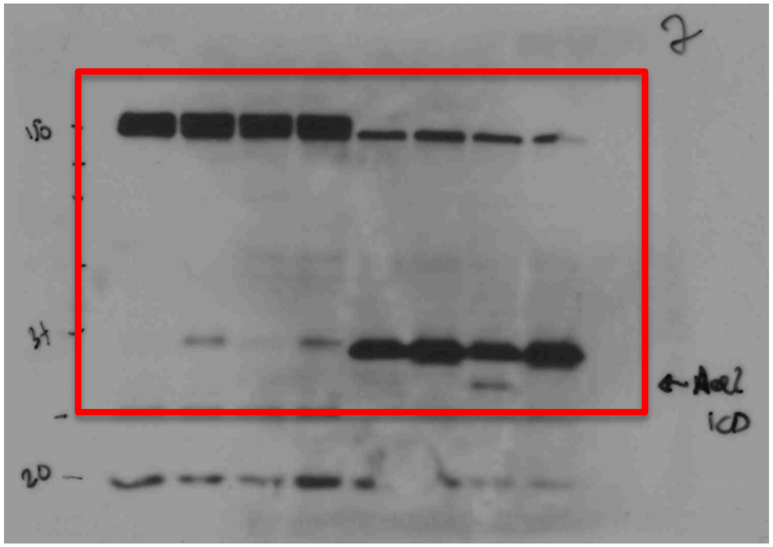

β-actin

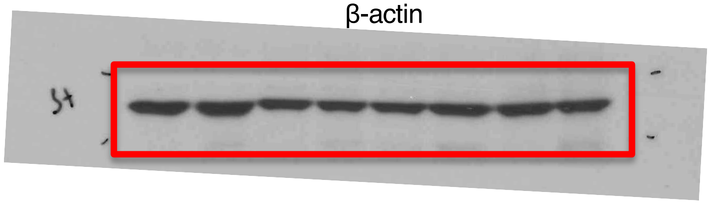

TMPRSS2

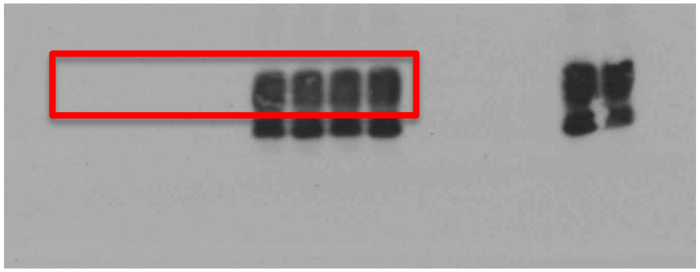

Fig1F

ACE2-GFP

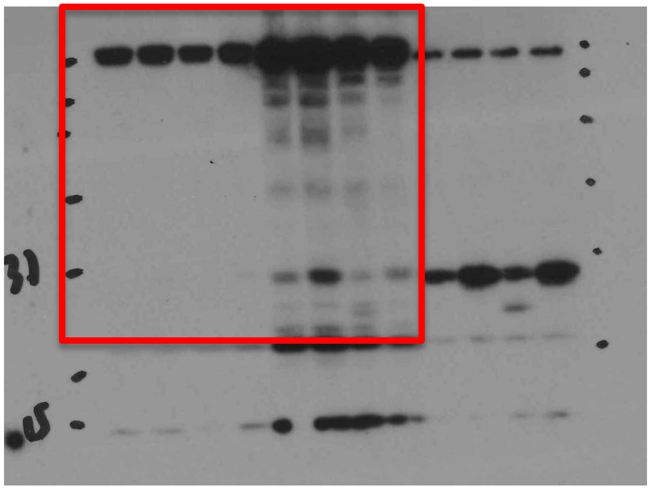

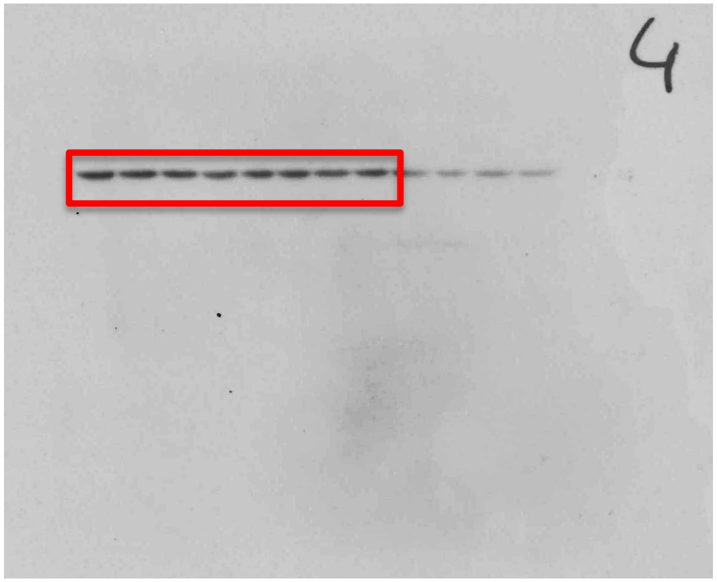

β-actin

Fig 1G, IP: C9

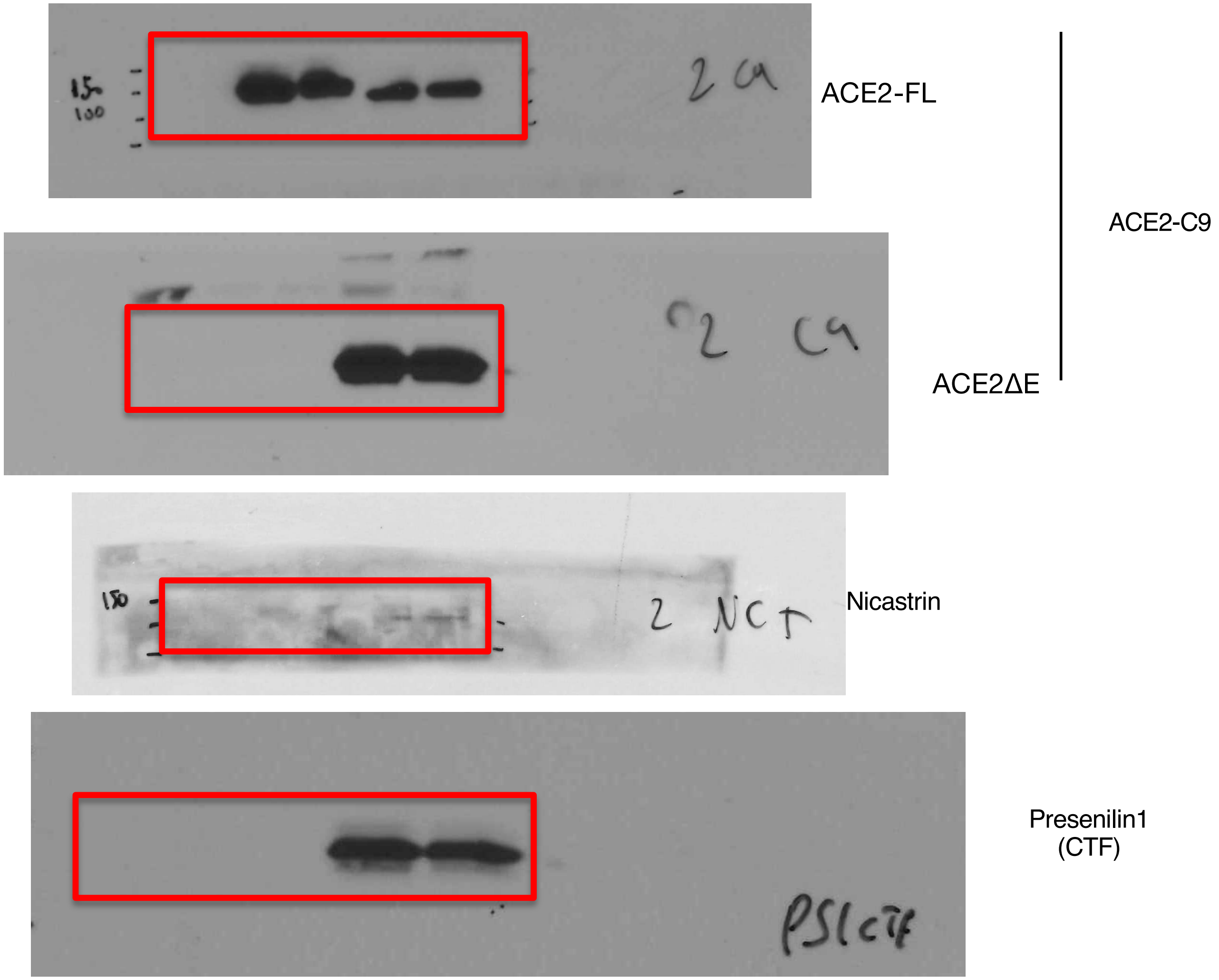

150
100
2 C9
ACE2-FL
ACE2ΔE
ACE2-C9
150
2 NCT
Nicastrin
Presenilin1
(CTF)
PS1 CTF

Fig 1G (Cont.) Total

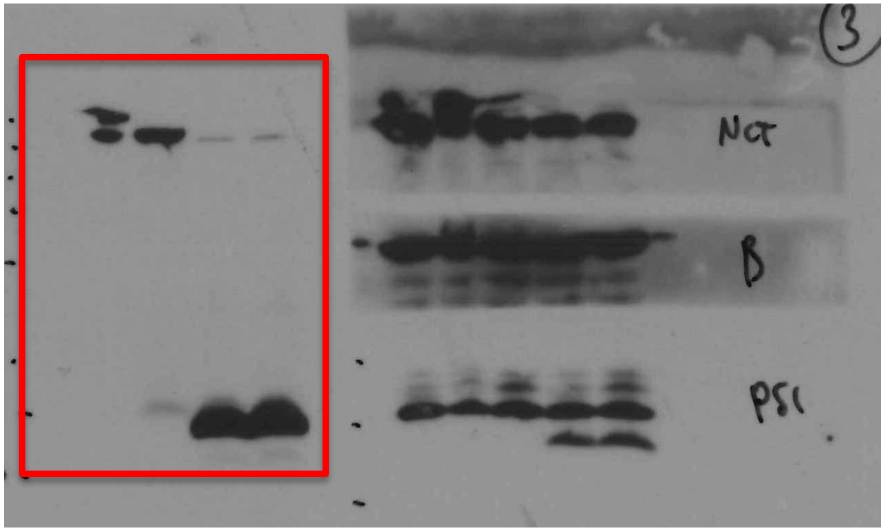

ACE2-C9

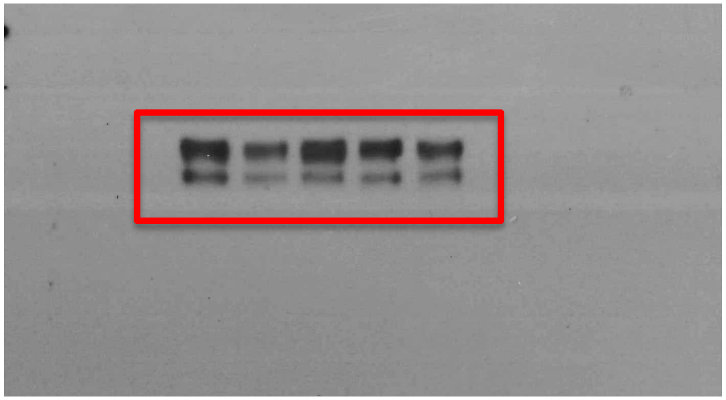

Nicastrin

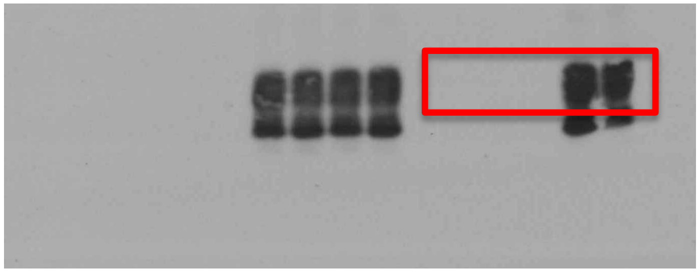

TMPRSS2

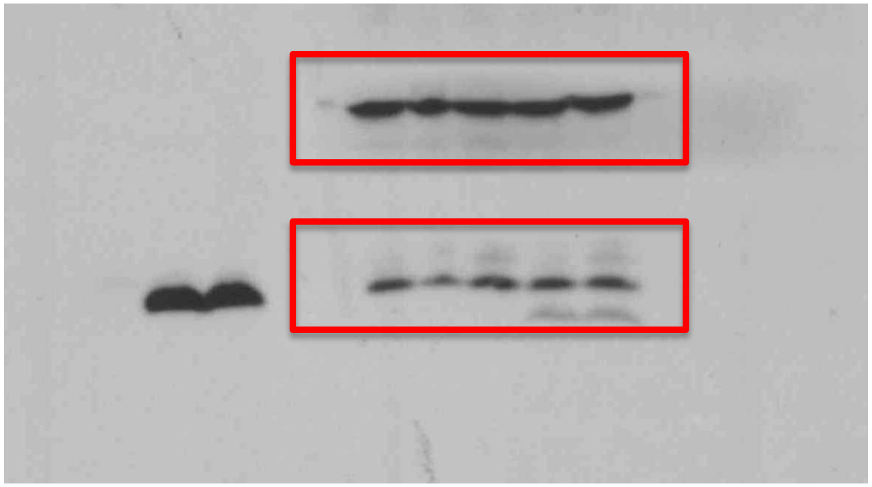

β-actin

Presenilin1 (CTF)

Fig 2A

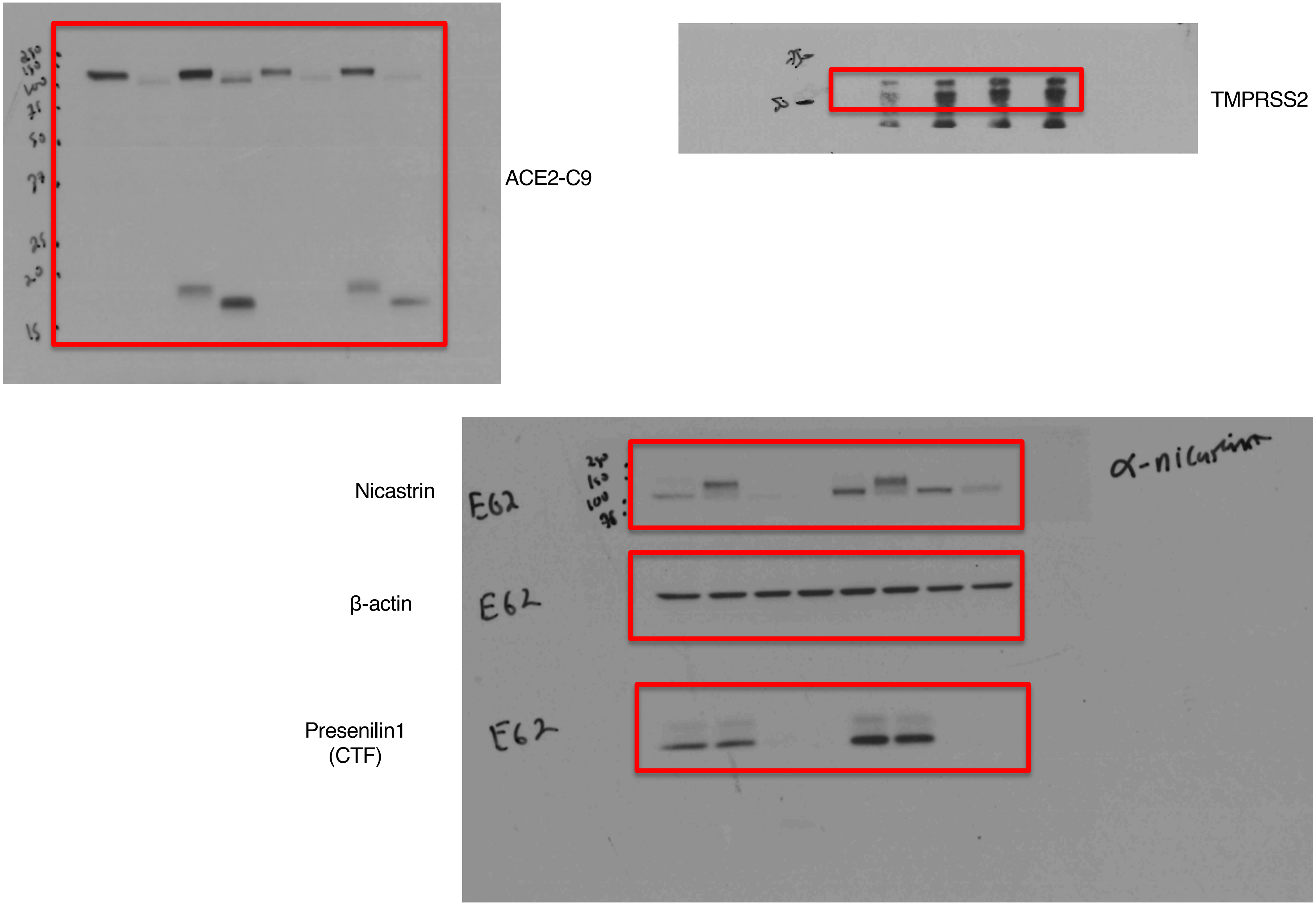
ACE2-C9
TMPRSS2
Nicastrin
E62
β-actin
E62
Presenilin1
(CTF)
E62

Fig3 A

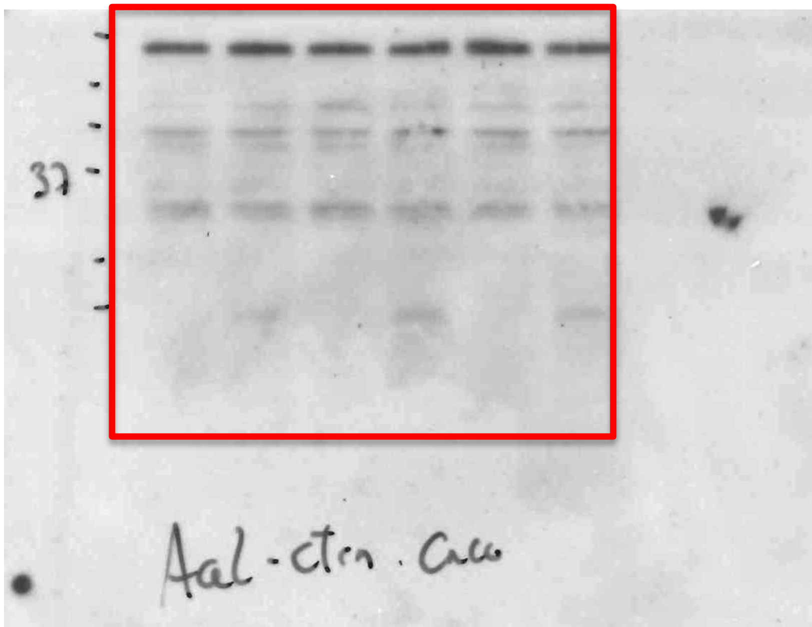

ACE2
(c-term)

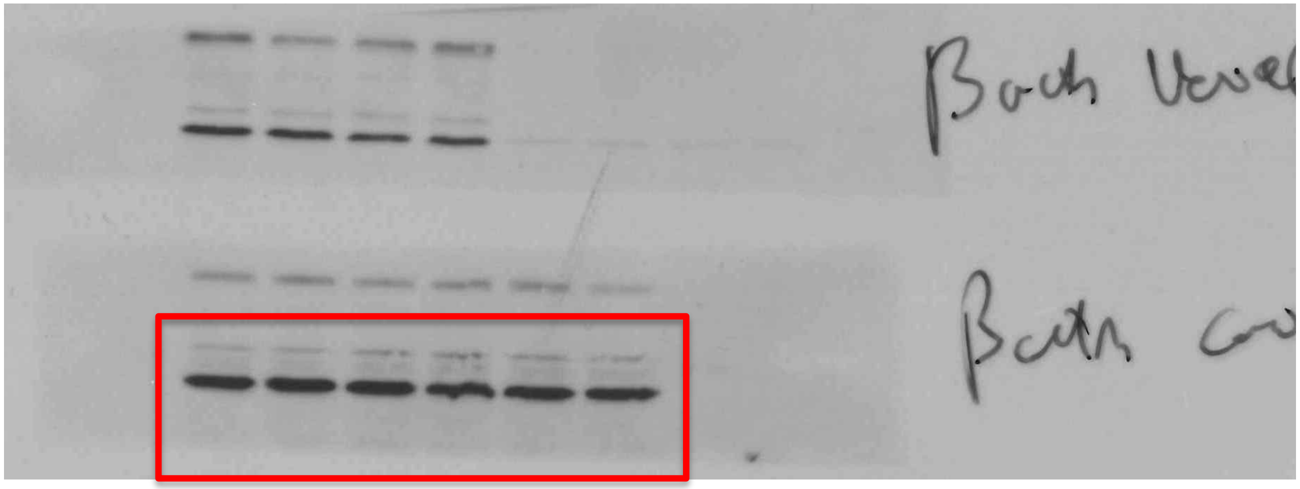

β-actin

Fig3 B

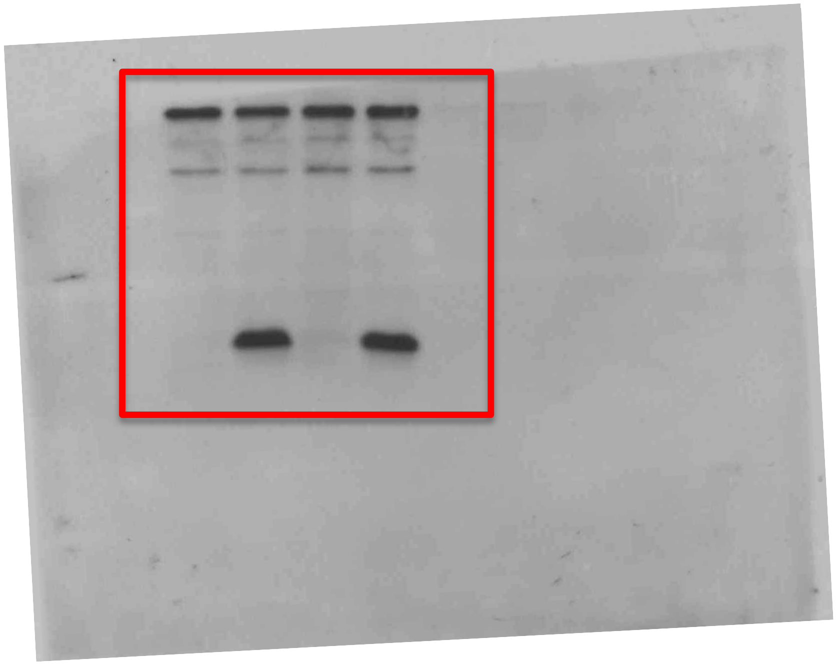

ACE2
(c-term)

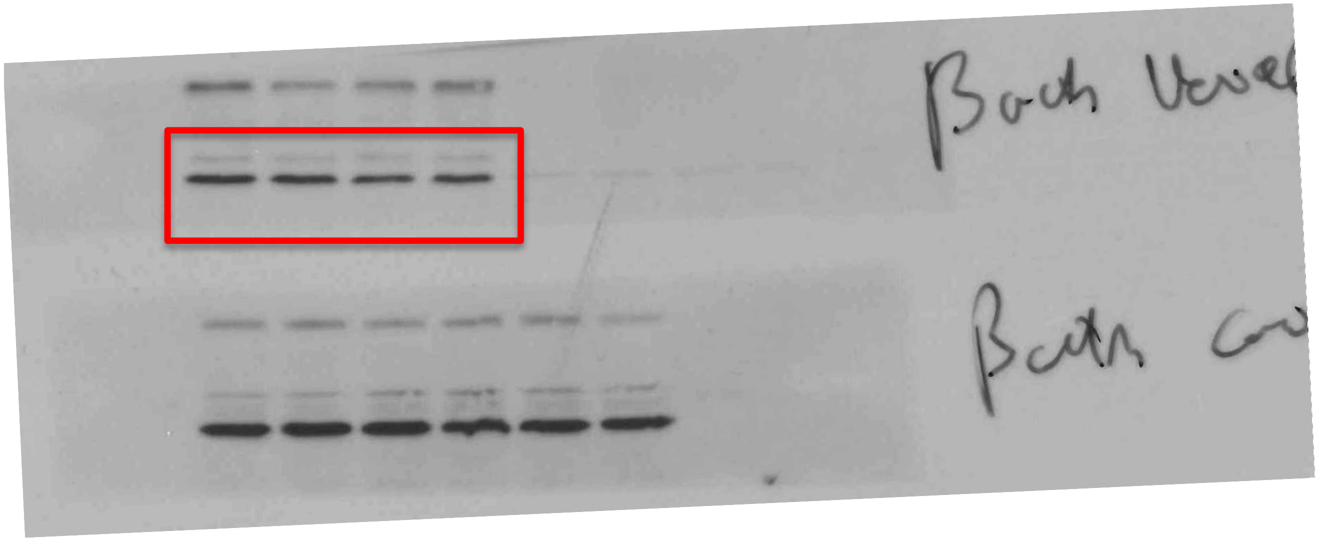

β-actin

Fig3 C

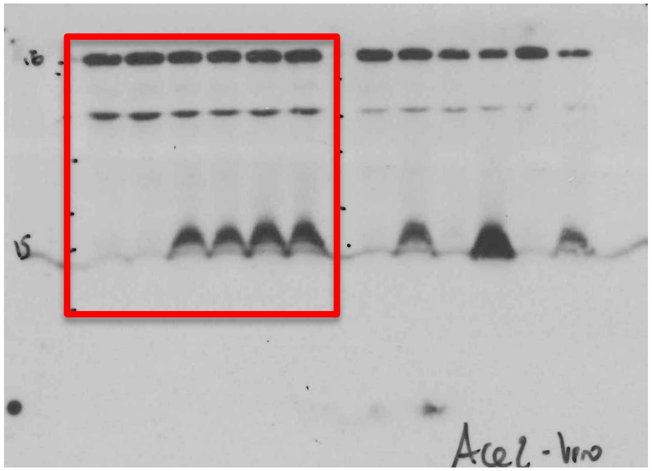

ACE2
(c-term)

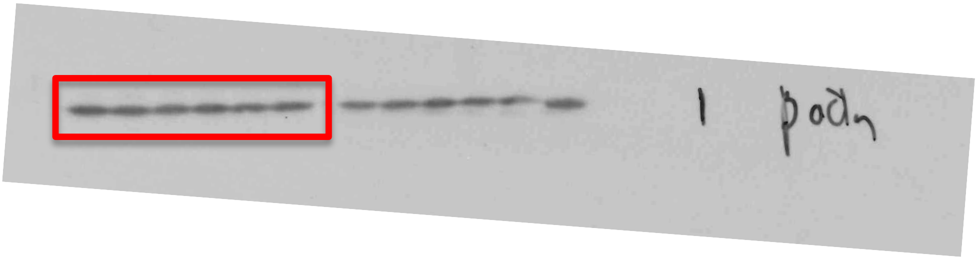

β-actin
